# Damage to epitaxial GaN layer on Al_2_O_3_ by 290-MeV ^238^U^32+^ ions irradiation

**DOI:** 10.1038/s41598-018-22321-w

**Published:** 2018-03-07

**Authors:** L. Q. Zhang, C. H. Zhang, J. J. Li, Y. C. Meng, Y. T. Yang, Y. Song, Z. N. Ding, T. X. Yan

**Affiliations:** 1 Institute of Modern Physics, Chinese Academy of Sciences, Lanzhou, 730000 China; 2 University of Chinese Academy of Sciences, Beijing, 100049 China

## Abstract

Micro-structural characteristics and electrical properties of an *n*-type GaN epilayer on Al_2_O_3_ irradiated by 290-MeV ^238^U^32+^ ions to various fluences were investigated using atomic force microscopy (AFM), scanning electron microscopy (SEM), high-resolution X-ray diffraction (HRXRD), and Raman scattering spectroscopy. AFM images show that the nano-hillocks generated, and the diameter and density of the nano-hillocks, increase obviously with increasing ion fluence, accompanied by an increase in surface roughness. SEM images display that the Al, O, and C elements appear on the GaN surface, along with a spiral-like, layered volcanic-cone structure formed at the highest-fluence irradiation. HRXRD reveals that the dislocation density increases, as the lattices gradually expand, and that Ga_2_O_3_ was produced with increasing ion fluence. Raman scattering spectra show that no N and Ga vacancies were produced, the free-carrier concentration decreases, while its mobility first increases and then exhibits a significant reduction with increasing ion fluence.

## Introduction

Gallium nitride (GaN) is one of the most promising III-V semiconductors for optoelectronic device applications compared with GaAs and InP due to its wide direct energy band gap (3.39 eV at room temperature) and its excellent electrical, optical, thermal, and radiation-tolerant properties^1,2^. GaN-based materials also have a great potential for use in high-temperature, high-power, and high-frequency electronic devices, such as high-electron-mobility transistors (HEMTs), heterojunction field-effect transistors (HFETs), and heterojunction bipolar transistors (HBTs)^3–7^.

With the development of space technology and the nuclear industry, these devices are widely used for working in the aerospace, aviation, and reactor fields. Such devices inevitably suffer from both particles and electromagnetic radiation, such as protons, electrons, alpha particles, neutrons, gamma rays, and swift heavy ions (SHIs). The passage of SHIs in materials mainly produces either intensive excitation or ionization of target electrons due to energy deposition from the SHIs. A long cylindrical high-temperature, high-pressure region containing charged ions is transiently produced along the ions’ trajectory. A rapid energy transfer results in various effects in materials, such as defect creation, defect annealing, amorphization and recrystallization^8^. When SHIs pass through the GaN-based electronic devices, the damage not only induces micro-structural change, but also leads to a change in electrical properties.

There have been extensive studies on GaN irradiated by various SHIs^9–17^. Kucheyev *et al*.^9^ reported the formation of continuous nano-tracks in GaN by 200-MeV Au-ion irradiation. Kumar *et al*.^10,11^ reported the formation of Ga_2_O_3_ and smoothness on the GaN surface irradiated with MeV heavy ions of O, Au, and Ag. Karlušić *et al*.^12^ reported the simultaneous formation of nano-hillocks and nano-holes on the GaN surface with 92-MeV Xe-ion irradiation, whereas only nano-holes appear with 23-MeV I-ion irradiation. Mansouri *et al*.^13^ reported the formation of circular regions in GaN with 132-MeV Pb-ion irradiation. Zhang *et al*.^14^ reported that rapid damage accumulation and efficient erosion occur in GaN at a rather low value of electronic energy deposition by using 230-MeV Pb ions. Kumar *et al*.^15^ reported that 200-MeV Ag ions cause an increase in resistivity and a degradation in carrier mobility of GaN/Al_2_O_3_ film. Sonia *et al*.^16^ reported that MeV protons, C, O, Fe, and Kr ions induce a degradation in carrier mobility of AlGaN/GaN HFET devices with increasing ion fluence. Titov *et al*.^17^ reported that MeV O, Li, and Si ions lead to a decrease in the free-carrier concentration of GaN and InGaP materials. As the application of GaN-based devices in the space and reactor fields continues to grow and their size has been ceaselessly minimized, more information regarding defects responsible for degradation is needed, so that device design improvements can be made to maximize system performance. Moreover, it is difficult for electrical characterization of damaged GaN material to be measured via Ohmic contacts due to the wide band gap and to the surface damage^18^. Raman spectroscopy is a powerful technique for studying the local transport properties in polar semiconductors over very small volumes^19^.

In this work, we choose the 290-MeV ^238^U^32+^ ion, the heaviest ion found in nature, which possesses the highest electron energy loss in GaN materials, compared with other mentioned ions above (Au, Ag, Pb and Xe ions)^9–15^, to systematically investigate the surface morphology and composition, dislocation density, free-carrier concentration as well as the mobility changes of GaN/Al_2_O_3_ film by means of atomic force microscopy (AFM), scanning electron microscopy (SEM), high-resolution X-ray diffraction (HRXRD), and Raman scattering spectroscopy. Moreover, all the mechanisms underlying aforementioned changes are discussed.

## Experiment

**Figure 1:**
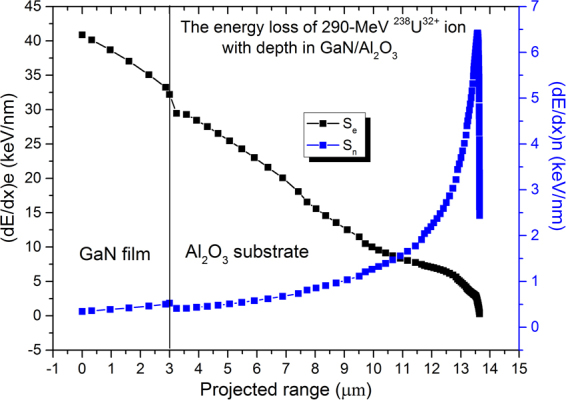
Variation of electron energy loss, *S*_e_, and nuclear energy loss, *S*_n_, as functions of the depth for 290-MeV ^238^U^32+^ ion in GaN/Al_2_O_3_.

An *n*-type wurtzite GaN layer is about 3 μm in thickness grown by MOCVD on *c*-plane sapphire substrates (supplied by Cree Inc., USA). Specimens with size of 10 × 10 mm^2^ were used for ^238^U^32+^ ion irradiation. The irradiation experiment was performed at a terminal chamber of the Sector Focused Cyclotron (SFC) in the National Laboratory of Heavy Ion Research Facilities in Lanzhou (HIRFL) at room temperature (RT). Vacuum pressure of the terminal chamber was 10^−7^ mbar. The kinetic energy of the ^238^U^32+^ ion was 290 MeV, and the fluence of the ^238^U^32+^ ion ranged from 1.0 × 10^9^ to 1.0 × 10^12^ ions/cm^2^. The flux of the ion beam was maintained as low as 5.0 × 10^8^ ions/cm^2^/s and the corresponding beam intensity was 3 nA in the irradiation experiment. SRIM 2006 code was used to calculate the depth distribution and energy deposition of the incident ions. For 290-MeV ^238^U^32+^ ion in GaN/Al_2_O_3_, the electron energy loss (S_e_), nuclear energy loss (S_n_) and the projected range were calculated as 40.88 keV/nm, 3.44 × 10^−1^ keV/nm, and ~13.7 μm, respectively. Hence, the ^238^U^32+^ ion loses its energy predominantly in the electronic stopping power regime throughout the entire ~3 μm-thick GaN film, and the ion end of range region is deep inside the sapphire substrate (~13.5 μm), as shown in Fig. 1.

After ^238^U^32+^ ion irradiation, the pristine and irradiated GaN samples were analyzed using tapping-mode AFM, SEM, HRXRD, and Raman scattering spectroscopy. The AFM used in this study is a model Veeco Dimension 3100, with a maximal operation voltage of 10 V and a scan frequency of 1.0 Hz. The model of the needle tip is HA-NC. The scan range is 40 μm × 40 μm and the scan scope along the *z*-axis is 100 nm. The SEM is a model of Nano-SEM 450 equipped with a field-emission gun with a maximal acceleration voltage of 35 kV. Electron image scanning and energy dispersive spectrometer (EDS) line scanning with an electron energy of 10 keV were carried out at a high-vacuum mode. The HRXRD measurements were performed using a D8 Discover X-ray diffractometer equipped with a four-crystal monochromator in Ge (220) configuration and one or two 200-μm slits before the detector. Monochromatic Cu *K*α_1_ X-rays (λ = 0.15406 nm) are used as the incident light. The ω-2θ and ω scans on the GaN (0002) crystal planes were done on all samples. Raman spectrum measurements were carried out using a JY-HR 800 micro-Raman system with a confocal microscopy system. The spectra were obtained at room temperature with the 632.8-nm excitation of a He-Ne laser in a backscattering geometry where the scattered light propagates in a direction parallel to the *c* axis of the GaN epilayer. The laser beam focused on the sample surface was about 2 μm in diameter. To avoid laser heating and local annealing effects, the He-Ne laser power was kept below 2 mW. The spectral resolution was 0.65 cm^−1^.

## Results and Discussion

### Surface morphology and composition analysis

**Figure 2:**
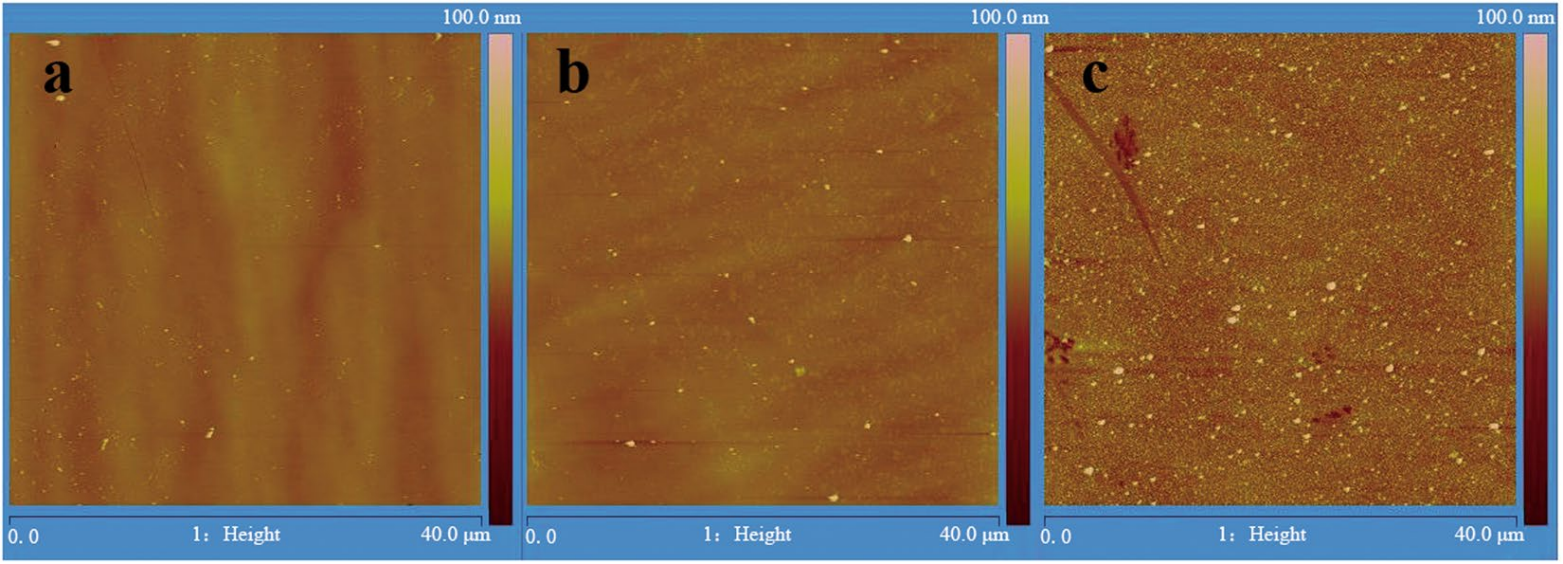
Typical 2D AFM images of the GaN film irradiated with 290-MeV U^32+^ ions at different fluences. Images a, b, and c show the surface morphologies of 1.0 × 10^10^, 1.0 × 10^11^ and 1.0 × 10^12^ ions/cm^2^ irradiation, respectively.

**Figure 3:**
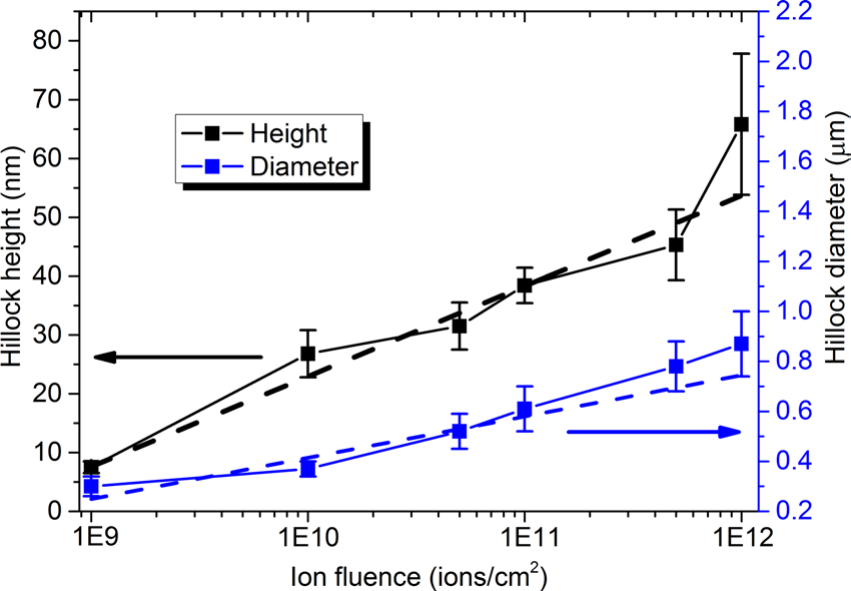
Hillock height and diameter as functions of the ion fluence after GaN films were irradiated with 290-MeV U^32+^ ions to different fluences.

Figure 2 shows typical 40 × 40 μm^2^ two-dimensional (2D) AFM images of the GaN samples irradiated with 290-MeV U^32+^ ions. Images a, b, and c show the surface morphologies of 1.0 × 10^10^, 1.0 × 10^11^ and 1.0 × 10^12^ ions/cm^2^ irradiation, respectively. After U^32+^ ions irradiation, nano-hillock-like defects grow out from a representative dislocation-mediated surface, which is made up of an array of terraces separated by about 2–3 nm high steps. With increasing ion fluence, the density, diameter and height of the nano-hillock-like defects obviously increase, as shown in Figs 2 and 3. The slope of the dashed lines approximately highlights the growth rate of the hillock height and diameter, based on linear fittings (Fig. 3). The average growth rate of the height and diameter is about (15.36 ± 0.93) nm/logΦ and (0.17 ± 0.03) µm/logΦ, respectively, (Φ is ion fluence) (Fig. 3). Meanwhile, the surface roughness also evidently increases with increasing ion fluence and the root-mean square (RMS) roughness of the irradiated sample is 9.3 nm (1.0 × 10^10^), 14 nm (1.0 × 10^11^) and 36.1 nm (1.0 × 10^12^), respectively. Furthermore, at the highest fluence (1.0 × 10^12^ ions/cm^2^) irradiation, the irradiated GaN surface is covered with a large number of nano-hillocks, cracks, and exfoliations that also appear, as well as the original surface morphology of GaN film vanishes. Similar experimental phenomena were also observed by Kumar *et al*.^11^.

According to the thermal spike model, the energy is first deposited by projectile ions in the electronic subsystem of the target. This energy is shared among the electrons by electron-electron coupling, and is subsequently transferred to the lattice atoms via electron-lattice interactions, leading to a rapid increase in the temperature and pronounced lattice heating along and in the vicinity of the ion path. The formation of surface hillocks can be ascribed to a melting process^20^. Moreover, a cylindrical zone of the order of several nanometers in diameter will form along the ions’ path, with high atomic mobility and reduced density. Therefore, this irradiated region may be strikingly different from the pristine. This effect of the high temperature and high pressure on the surrounding material, and also on the direct modification of the material in the track itself, can create new potential gradients, which act as a driving force for further modification and create nanostructures during ion irradiation^21^. In order to further detect the compositions and the microstructure of the hillocks on irradiated GaN surfaces, we analyze the hillocks in detail using SEM.

**Figure 4:**
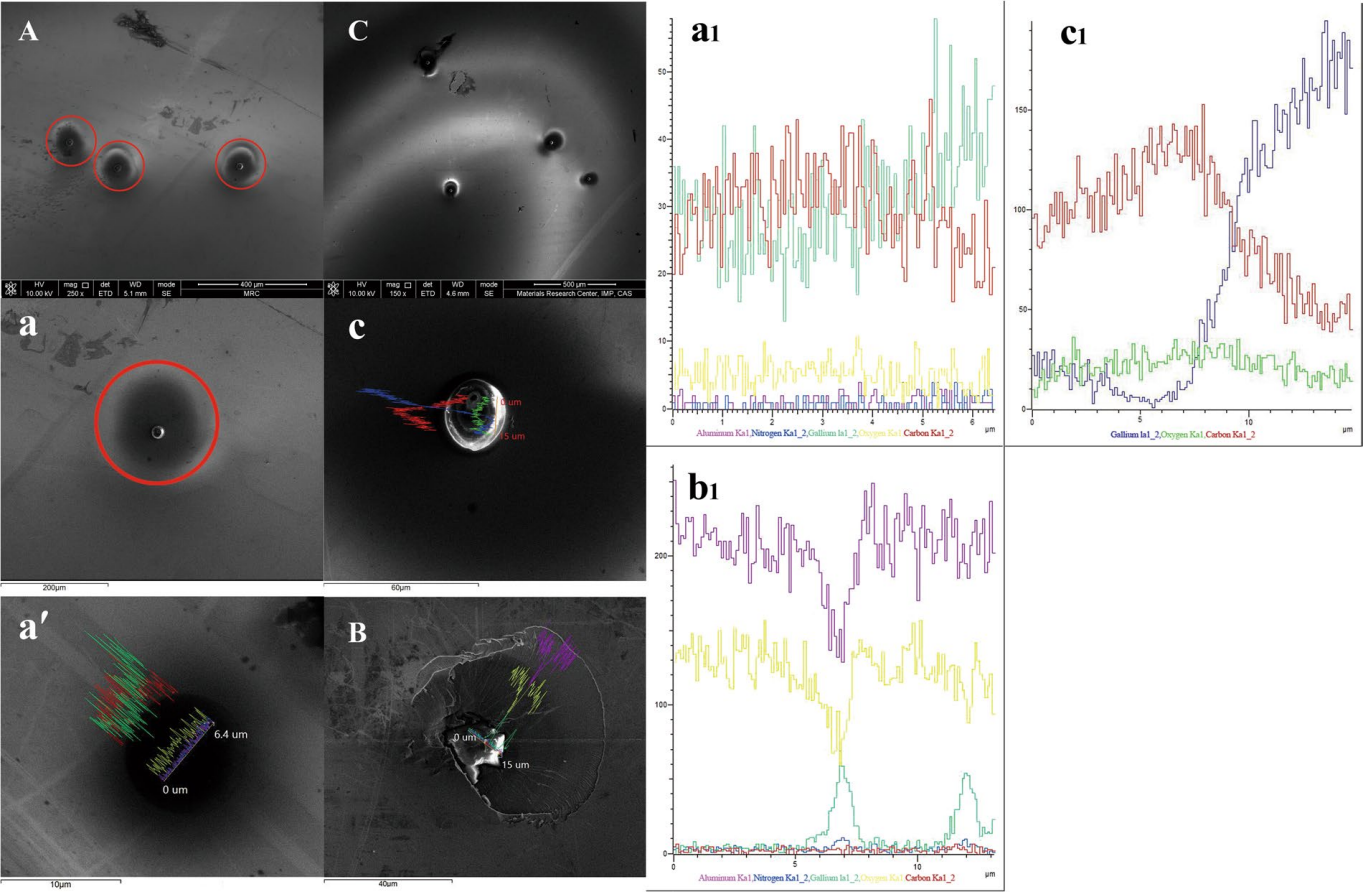
SEM images of hillock morphology and composition distribution on GaN surface after irradiation at different fluences. (**A**–**C**) are electron images for 1.0 × 10^10^, 1.0 × 10^11^ and 1.0 × 10^12^ ions/cm^2^ irradiation, respectively. Images a and c are an enlargement of the hillocks in images A and C, respectively. Image a' is an enlargement of the central region of image a. (**a**_**1**_,**b**_**1**_,**c**_**1**_) are the EDS line-scan profiles of a hillock along the diameter corresponding to a', B and c.

Figure 4 shows the compositions and microstructure of hillocks on irradiated GaN surfaces at different fluences. For lower-fluence (1.0 × 10^10^ ions/cm^2^) irradiation, a hillock is observed, surrounded by a faint halo-like damaged zone with a diameter of about 150 µm (*r*~*v*^2^)^22^, in addition to a pit on the hillock center with a diameter of about 9 µm (*r*~*v*)^22^, as shown in images A, a and a′. This defect halo was formed by spatial spreading of valence holes^22^.

With increasing ion fluence, the molten atoms spread across the surface, after they were poured out along the ions’ trajectory, as shown in image B of Fig. 4. As the ion fluence increases further, a spiral-like layered hillock with a diameter of about 30 µm, resembling a volcanic-cone structure, was formed, as shown in images C and c. Generally speaking, as the fluence increases, the diameter of the hillock also rapidly increases. However, except for Ga and N, the O, C, and Al elements also appear around and on a hillock (Figs. 4a_1_,b_1_ and c_1_), indicating that the sapphire (Al_2_O_3_) substrate decomposes into Al and O at high temperature. Moreover, molten Al and O atoms diffuse into GaN along the heated trajectory, and subsequently they were pushed onto the GaN surface by extrusion force from the undamaged lattice through the ion path. In addition, note that the N content is very low on the irradiated surface; N loss was also observed in our previous experiment^23^.

### Microstructure and electrical properties analysis

**Figure 5:**
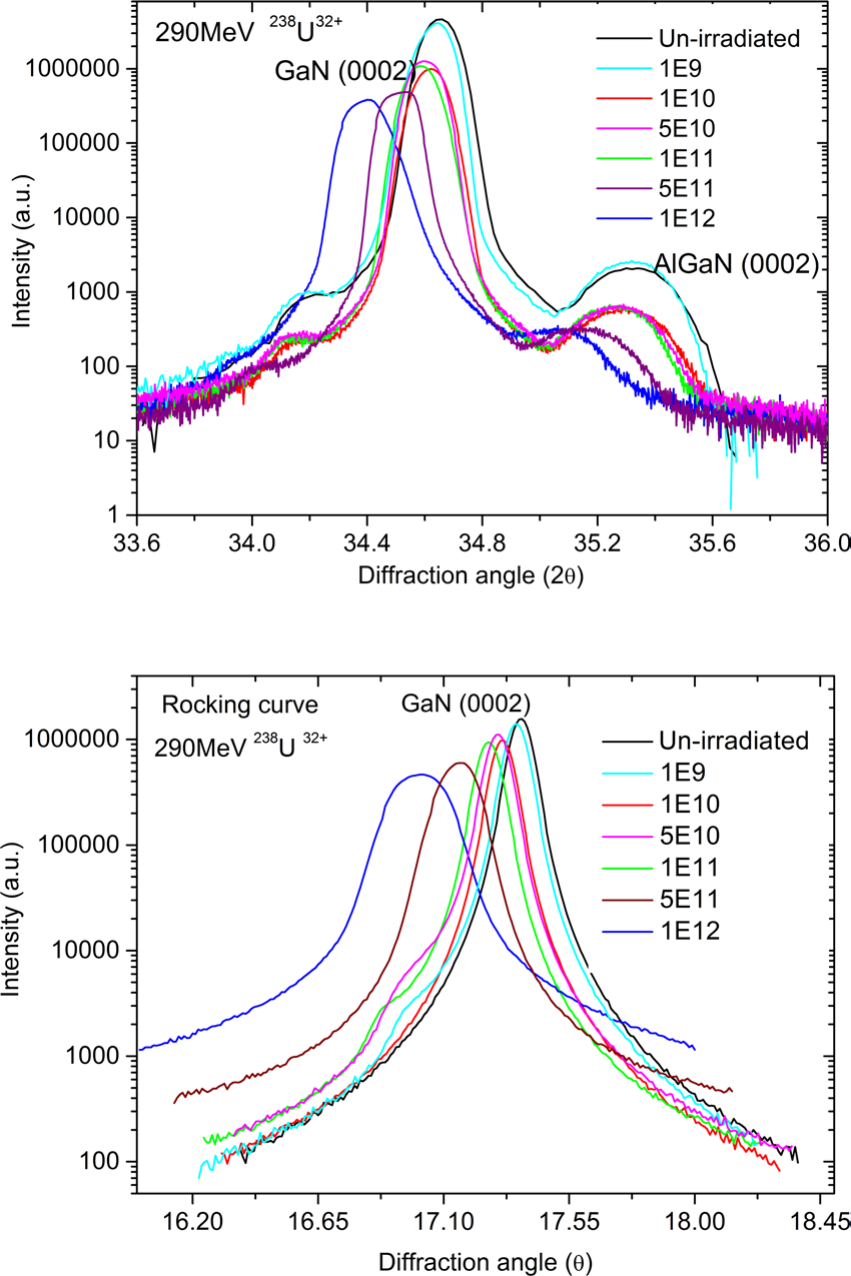
GaN (0002) HRXRD patterns and rocking curves after irradiation with 290-MeV U^32+^ ions at fluences from 1.0 × 10^9^ to 1.0 × 10^12^ ions/cm^2^.

Figure 5 shows the high-resolution X-ray-diffraction patterns and rocking curves of a GaN (0002) peak after irradiation with 290-MeV U^32+^ ions at fluences from 1.0 × 10^9^ to 1.0 × 10^12^ ions/cm^2^. (ω-2θ) and ω scan patterns of the GaN (0002) peak were performed, separately. According to standard XRD patterns of AlN (JCPDS, Card, No. 08–0262) and GaN (JCPDS, Card, No. 02–1078), the un-irradiated sample exhibits a sharp GaN (0002) peak and a broad AlGaN (0002) peak located at 2θ = 34.65° and 35.32°, respectively. This observation is generally consistent with the results reported in the literatures^10,24^. After irradiation, the intensity of the GaN (0002) and the AlGaN (0002) peaks decreases generally with increasing incident ion fluence. The original diffraction peaks of the GaN (0002) and the AlGaN (0002) lattice shift towards smaller angle and the full width at half-maximum (FWHM) of the GaN is found to increase from 0.14° to 0.17° with increasing ion fluence, indicating that the expansion and distortion in GaN and AlGaN crystal lattices occur, in addition to compressive stress produced along the ion incident direction. This shift and broadening of the GaN (0002) peak are mainly due to stress regions and the creation of various defects.

Meanwhile, as shown in Fig. 5, with increasing ion fluence, the FWHM of the (0002) crystal plane in the rocking curve graph also increases. This broadening of the FWHM of the (0002) plane in X-ray rocking curves is mainly induced by the rotation and tilt of lattice planes, as well as by strain fields for single-crystal GaN due to the production of dislocation loops and screw dislocations^25–27^. The 290-MeV U^32+^ ions pass through a 3-µm-GaN film and stop in the Al_2_O_3_ (Fig. 1). During this energy deposition process, high electron energy loss (40.88 keV/nm) not only can induce latent tracks and dislocation loops in GaN, but can also generate screw dislocations in GaN due to high temperature and high stress. The latter was perhaps embodied from cracks, layered hillocks and surface compositions observed from AFM and SEM images (Figs 2 and 4). Similar phenomena were observed by several groups in SHI-irradiated III-V nitrides via TEM, AFM and HRXRD techniques^9,13,26,27^.

According to the equation^28^

1 $${\rho }_{s}=\frac{{\beta }^{2}(0002)}{2\pi .\,\mathrm{ln}\,2\times {|{b}_{c}|}^{2}},$$

where, *ρ*_*s*_ is the dislocation density, *β*(0002) the FWHM of the (0002) crystal plane in the rocking curve graph, and *b*_*c*_ the Burgers vector parallel to the *c* axis. For a hexagonal GaN, *b*_*c*_ = 0.5185 nm^29^. we can obtain the dislocation density at different fluences. The variation of the dislocation density with the fluence of 290-MeV U^32+^ ions is given in Table 1. The dislocation density initially increases very slowly up to a fluence of 1 × 10^11^ ions/cm^2^. Above this fluence, it increases rapidly with respect to fluence; this drastic increase was interpreted as being caused by mutual spatial tangles and penetrations between the dislocations. The dislocation production is mainly ascribed to the release of the strain due to lattice expansion and defect creation.

**Table 1:** Dislocation density of GaN (0002) crystal plane after irradiation with U^32+^ ions to different fluences.

Fluence (ions/cm^2^)	Un-irradiated	1.0 × 10^9^	1.0 × 10^10^	5.0 × 10^10^	1.0 × 10^11^	5.0 × 10^11^	1.0 × 10^12^
FWHM (arcsec)	322.2	328.1	335.8	341.5	342.9	557.5	859.5
Dislocation density (10^8^.cm^−2^)	2.087	2.159	2.261	2.339	2.357	6.230	14.80

**Figure 6:**
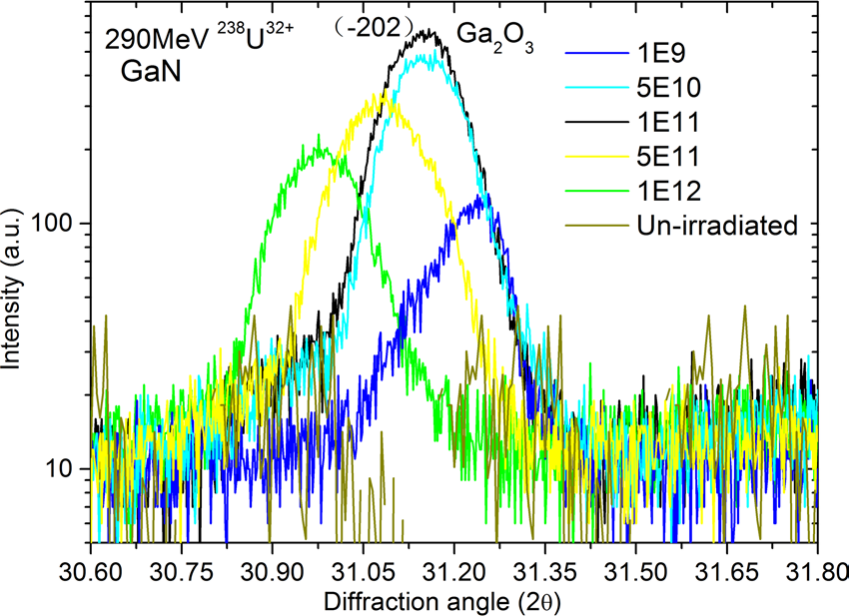
Ga_2_O_3_ (−202) HRXRD pattern of 290-MeV U^32+^ ions-irradiated GaN/Al_2_O_3_ with fluences from 1.0 × 10^9^ to 1.0 × 10^12^ ions/cm^2^.

Figure 6 shows the β-Ga_2_O_3_ X-ray diffraction pattern of 290-MeV U^32+^ ions-irradiated GaN/Al_2_O_3_ with fluences from 1.0 × 10^9^ to 1.0 × 10^12^ ions/cm^2^. The peak 2θ~31.15° is attributed to the β-Ga_2_O_3_ (−202) diffractive peak^30^. The formation of Ga_2_O_3_ is due to the dissociation of Al_2_O_3_ and the mixture of Al_2_O_3_/GaN^31^. Moreover, the diffraction peak of the Ga_2_O_3_ lattice shifts towards smaller angle with increasing ion fluence. Similarly, Kumar *et al*.^32^ reported the mixing of Al_2_O_3_/GaN using 100-MeV Ni^9+^ ions. Sarvesh *et al*.^33^ reported the mixing of Cu/Ge bilayers due to the irradiation with 120-MeV Ag ions. The formation of Ga_2_O_3_ may be useful to understand the role of electrical transport behavior in semiconductor/substrate materials^32^.

**Figure 7:**
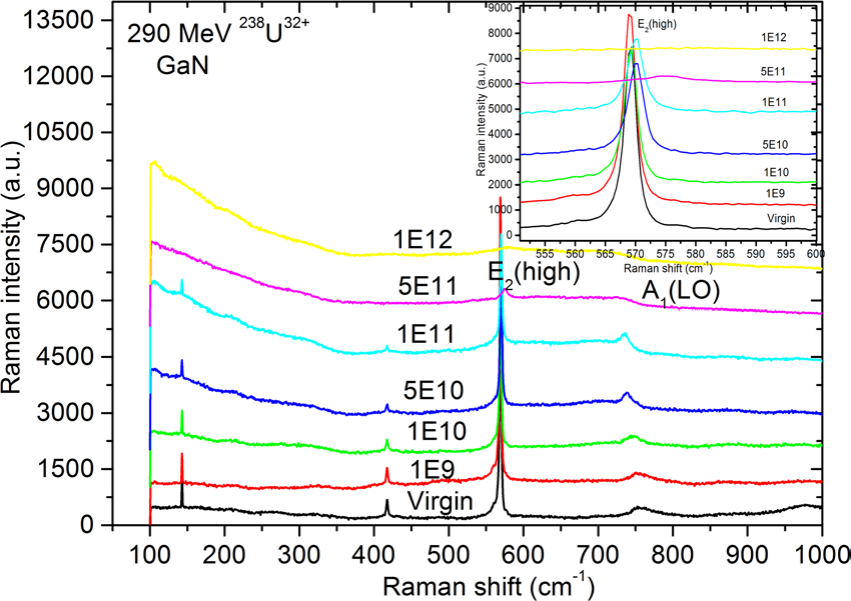
Room temperature Raman scattering spectra of the GaN film with 290-MeV U^32+^-ion irradiation at different fluences. Inset shows amplified *E*_2_ (high) phonon energy of irradiated GaN film as a function of the fluence. All spectra are shifted vertically for clarity.

Figure 7 shows the room temperature Raman scattering spectra of the pristine and the GaN epilayer irradiated with 290-MeV U^32+^ ions at different fluences. In the spectra of all the samples, the peaks near 144, 570, 740 cm^−1^ are the first-order phonon frequencies of the *E*_2_ (low), *E*_2_ (high), and *A*_1_ [longitudinal optical (LO)] phonon, respectively^34,35^, reflecting the characteristics of the hexagonal-crystal-phase GaN. After irradiation with successively increasing fluence, the strain-sensitive *E*_2_ (high) phonon band blueshifts and broadens in FWHM generally, indicating that the irradiated GaN samples experience compressive stress along the *z* axis (//*c*)^36^, matching with the results obtained from HRXRD measurements. Furthermore, such a blueshift also indicates that the length of the Ga-N bond is shortened, the bond energy of the Ga-N increases, and defects are generated. When the fluence increases to 1.0 × 10^12^ ions/cm^2^, both the *E*_2_ (high) and *A*_1_ (LO) phonon modes completely disappear, indicating that the violation of Raman selection rules in this backscattering geometry occurs due to surface-specific tilt or twist caused by strain relief. Meanwhile, a slope-like feature between 100 and 200 cm^−1^ is observed for the highest fluence irradiation (1.0 × 10^12^ ions/cm^2^), because GaN lattices are disordered, which leads to a loss of translational periodicity and, hence, the relaxation of *q* = 0 conservation. In addition, the peak at 417 cm^−1^ entirely disappears for the highest fluence irradiation (1.0 × 10^12^ ions/cm^2^), indicating that this peak is a vibration mode of the N-rich Ga-N bond configuration, and does not come from the sapphire substrate^37^. Compared with the Raman spectra of GaN implanted by various ions with different fluences reported elsewhere^36,38,39^. The peaks at 300 and 670 cm^−1^ attributed to disorder-activated Raman scattering (DARS) related to Ga and N vacancies did not arise in our measurements, because O and Al occupy the vacancies of N and Ga due to the sapphire substrate decomposition. Furthermore, the O impurities obstruct the preferential removal of N atoms due to the larger electronegativity of O atoms^40^. In addition, the projected range of U^32+^ ions is much larger than the thickness of GaN films, and the irradiation resulted in an almost homogeneous energy deposition in the GaN film mainly through the intense electronic energy deposition process in this experiment, as shown in Fig. 1. There are no Raman peaks from Ga_2_O_3_ due to the non-fulfillment of Raman selection rules under this scattering geometry.

**Figure 8:**
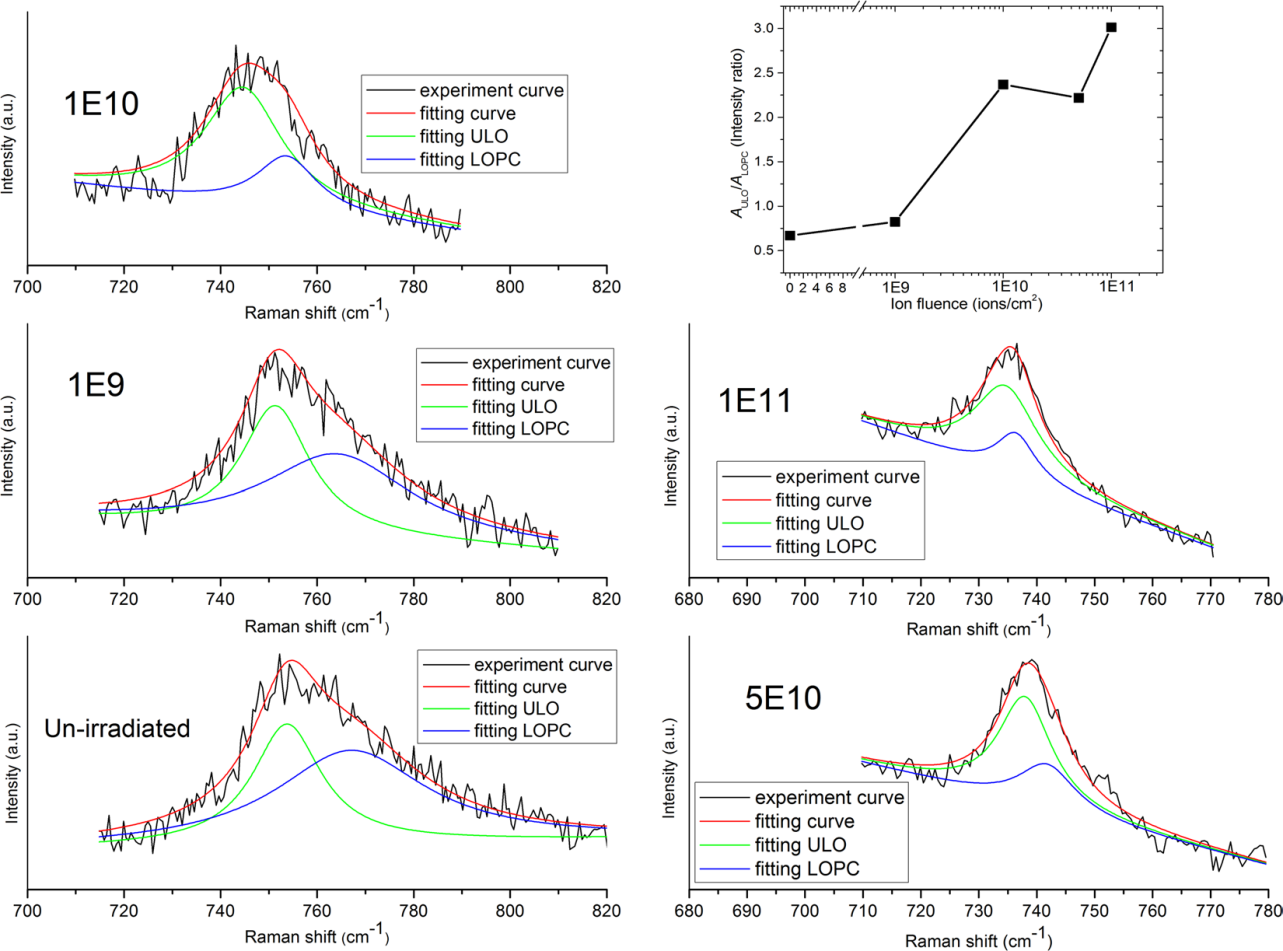
ULO and LOPC peaks decomposed from *A*_1_(LO) phonon bands of GaN film with 290-MeV U^32+^-ion irradiation at different fluences. Black lines are original experiment curves, red lines are the fitting curves of original experiment data, and green lines and blue lines represent the ULO and LOPC modes, respectively. Green lines and blue lines are decomposed curves of the red lines using Lorentz fitting. Inset:Area ratio of the ULO to LOPC peaks at different fluences.

The *A*_1_ (LO) phonon band is composed of the unscreened-LO mode originating in the surface depletion layer and the LO phonon-plasmon coupled mode^41^. Similar studies were reported by several groups^42–44^. For *n*-type GaN, acceptors on the surface capture the electrons from the conduction band, forming a space charge region near the surface with positive charges and producing an electric field towards the inside from the surface. This leads to *n*-type GaN’s energy band bending upwards near the surface, deviating from the Femi energy level. Therefore, the electron concentration is lower near the surface compared to that of the inside. Thus, a surface depletion layer is formed. Plasmon polaritons and free carriers nearly do not exist in the depletion layer. The *A*_1_ (LO) phonon band broadens with asymmetric line shape, because of the phonon confinement effect and the broken longitudinal symmetry caused by a high surface-to-volume ratio due to the formation of nano-hillocks at the surface^45^. In order to extract the electrical information from a coupled LO phonon-damped plasmon mode, we separated the *A*_1_ (LO) phonon band into the unscreened-LO peak (ULO) and LO phonon-plasmon coupled peak (LOPC), and plotted the values of the *A*_ULO_/*A*_LOPC_ area ratio versus ion fluence, as shown in Fig. 8. With increasing ion fluence, the area of the unscreened LO peak increases generally, indicating that the surface depletion zone thickness increases continuously due to the growing surface damage^46^, which is in good agreement with AFM observations.

**Figure 9:**
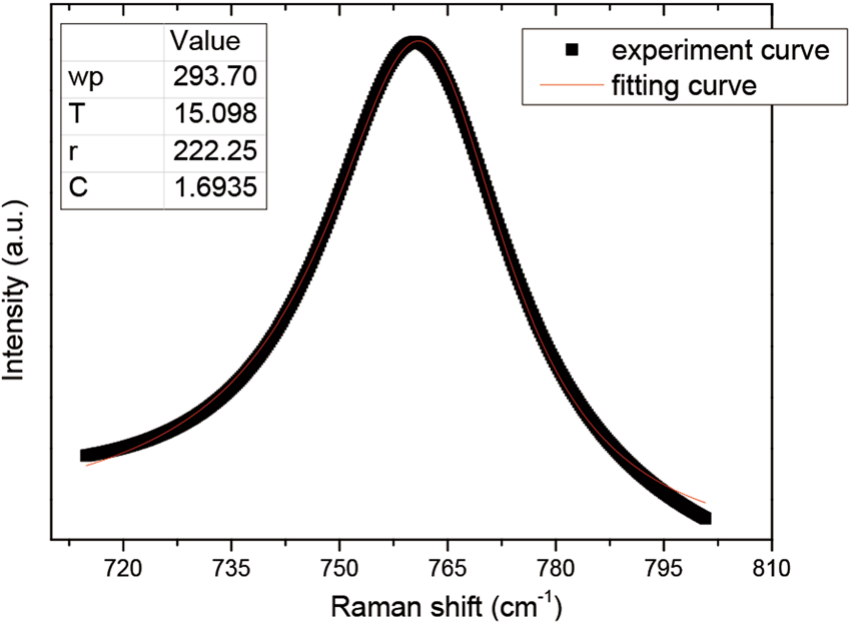
Comparison of experimental and fitting line shapes of the LOPC peak for 1 × 10^9^ ions/cm^2^ irradiation. All LOPC experimental curves were fitted in the same way.

The scattering mechanisms of the LOPC peak separated from the *A*_1_(LO) phonon band are the LO phonon-plasmon coupled modes. For a hexagonal crystal, the Raman intensity profile is expressed by^47^

2 $${I}_{A}=\frac{{d}^{2}S}{d\omega d{\rm{\Omega }}}|{}_{A}=SA(\omega ){\rm{Im}}(-\frac{1}{\varepsilon }),$$

$$\begin{array}{rcl}A(\omega ) & = & 1+2C\frac{{\omega }_{t}^{2}}{{\rm{\Delta }}}[{\omega }_{p}^{2}\gamma ({\omega }_{t}^{2}-{\omega }^{2})-{\omega }^{2}{\rm{\Gamma }}({\omega }^{2}+{\gamma }^{2}-{\omega }_{p}^{2})]+{C}^{2}(\frac{{\omega }_{t}^{4}}{{\rm{\Delta }}({\omega }_{l}^{2}-{\omega }_{t}^{2})})\\  &  & \{{\omega }_{p}^{2}[\gamma ({\omega }_{l}^{2}-{\omega }_{t}^{2})+\Gamma ({\omega }_{p}^{2}-2{\omega }^{2})]+{\omega }^{2}{\rm{\Gamma }}({\omega }^{2}+{\gamma }^{2})\},\\ {\rm{\Delta }} & = & {\omega }_{p}^{2}\gamma [{({\omega }_{t}^{2}-{\omega }^{2})}^{2}+{(\omega {\rm{\Gamma }})}^{2}]+{\omega }^{2}{\rm{\Gamma }}({\omega }_{l}^{2}-{\omega }_{t}^{2})({\omega }^{2}+{\gamma }^{2}),\\ \varepsilon (\omega ) & = & {\varepsilon }_{\infty }(1+\frac{{\omega }_{l}^{2}-{\omega }_{t}^{2}}{{\omega }_{t}^{2}-{\omega }^{2}-i\omega {\rm{\Gamma }}}-\frac{{\omega }_{p}^{2}}{\omega (\omega +i\gamma )}),\end{array}$$

3 $${\omega }_{p}^{2}=\frac{4\pi n{e}^{2}}{{\varepsilon }_{\infty }{m}^{\ast }},$$

4 $$\gamma =\frac{e}{{m}^{\ast }\mu },$$

where, for GaN, ${\omega }_{\ell }$ and *ω*_*t*_ are the frequencies of LO and TO phonon, ${\omega }_{\ell }$ = 735 cm^−1^ and *ω*_*t*_ = 533 cm^−1^, respectively; *ε* is the dielectric function; and ${\varepsilon }_{\infty }$ is the high-frequency dielectric constant, ${\varepsilon }_{\infty }$ = 5.35. *e* is the electron charge; *γ* is the plasmon damping constant; Γ is the phonon damping constant; *ω*_*p*_ is the plasmon frequency; *n* is the free-carrier concentration; and m* is the effective mass of the free carrier, ${m}^{\ast }=0.2{m}_{e}$. *μ* is the free-carrier mobility, *ω* is the Raman frequency shift, *C* is the Faust-Henry coefficient^48^, and *C* and *S* are considered as constant coefficients. Taking *ω*_*p*_, *γ*, Γ, and *C* in Eq. (2) as fitting parameters, we fitted the LOPC peak line shape to obtain the *ω*_*p*_, *γ*, Γ, and *C* values. The coupled mode in each sample is normalized by the peak intensity. The fitting line shape of the LOPC peak for 1 × 10^9^ ions/cm^2^ irradiation is displayed in Fig. 9. LOPC peaks of all specimens were fitted in the same way. The values of *n* and *μ* are derived from the fitting parameters *ω*_*p*_ and *γ*, respectively, according to Eqs (3) and (4). The results of fitting and calculation are given in Table 2.

**Table 2:** LOPC-peak fitting results of the GaN film with U^32+^-ion irradiation at different fluences.

Fluence (ions/cm^2^)	*S*	${{\boldsymbol{\omega }}}_{{{\boldsymbol{p}}}_{{\boldsymbol{(}}c{{\bf{m}}}^{-{\bf{1}}}{\boldsymbol{)}}}}$	${{\boldsymbol{\gamma }}}_{{\boldsymbol{(}}c{{\bf{m}}}^{{\boldsymbol{-}}1}{\boldsymbol{)}}}$	${{\boldsymbol{\Gamma }}}_{{\boldsymbol{(}}c{{\bf{m}}}^{{\boldsymbol{-}}1}{\boldsymbol{)}}}$	*C*	${{\boldsymbol{n}}}_{{\boldsymbol{(}}1{{\bf{0}}}^{{\bf{17}}}{\bf{c}}{{\bf{m}}}^{{\boldsymbol{-}}3})}$	${{\boldsymbol{\mu }}}_{({\bf{c}}{{\bf{m}}}^{2}/{\bf{Vs}})}$
Un-irradiated	0.9982	316.51	261.53	10.154	0.3909	14.899	170.68
1.0 × 10^9^	0.9996	293.70	222.25	15.098	1.6935	12.829	200.85
1.0 × 10^10^	0.9985	246.31	178.076	14.093	1.5506	9.0229	250.67
5.0 × 10^10^	0.9979	145.95	147.32	20.732	1.4776	3.1680	302.99
1.0 × 10^11^	0.9978	86.122	980.16	13.698	1.3495	1.1031	45.540

As shown in Table 2, the free-carrier concentration decreases with increasing ion fluence. In *n*-type GaN, the free-carrier concentration *n* = N_*d*_−N_*a*_, and O_N_ and C_Ga_ are shallow donors^49^, while C_N_ is a more stable shallow acceptor^50^. Moreover, the charged dislocation is an acceptor-like defect^51^. The aforementioned decrease can mainly be attributed to the compensation effect due to the growing charged dislocations (acceptor-like defects) with increasing ion fluence, which was revealed by HRXRD. Furthermore, the difficult excitation of electrons in the valance band caused by an increase in the band gap due to Ga_2_O_3_ formation also accounts for this reduction in free-carrier concentration^52^. Table 2 also shows that the free-carrier mobility first increases and then exhibits a decrease with increasing ion fluence. The mobility of free carriers depends on various scattering mechanisms. For lower-fluence irradiation, the expansion of lattices results in a weakness from lattice vibration scattering. Moreover, the carrier mobility µ = µ_*d*_ + µ_*a*_, many donors (O_N_, C_Ga_)^49^ and acceptors (C_N_)^50^ were generated at lower-fluence irradiation. As the ion fluence increases, the charged dislocations dominate the scattering mechanism, which leads to a degradation in the carrier mobility^51^. Only when the distance between the charged dislocations is less than a certain value, can the dislocations be predominate with respect to the carrier mobility^53^.

## Conclusions

Irradiation with 290-MeV ^238^U^32+^ ions on MOCVD-grown *n*-GaN/Al_2_O_3_ epilayers was carried out at room temperature at fluences from 1.0 × 10^9^ to 1.0 × 10^12^ ions/cm^2^. The irradiated samples were characterized using AFM, SEM, HRXRD, and Raman scattering measurements. The mixture of GaN/Al_2_O_3_ is ascribed to the dissociation of Al_2_O_3_ substrate as a result of high electron energy loss of the U^32+^ ion. Hillock formation on the GaN surface is the representation of dislocation occurrence, and the density of both the hillock and the dislocation increases with increasing ion fluence. Moreover, dislocations remarkably affect the atoms and charges transport due to the stress field, electrostatic field and dangling bonds around the dislocation line. The significant amount of charged dislocation-defects produced enhance the trapping and scattering of the free carriers, leading to a reduction in free-carrier concentration and a general degradation in free-carrier mobility. Meanwhile, dislocations provide passages for Al and O elements to spread upward, promoting the generation of Ga_2_O_3_. In contrast to other experiments^15,22,54^, no N and Ga vacancies were generated, and the damage to GaN/Al_2_O_3_ from 290-MeV U^32+^ -ion irradiation is less than that of from other SHIs at the same fluence due to the incorporation of O and Al elements and Ga_2_O_3_ formation. These results suggest that the properties and performance of Ш-nitride semiconductor devices working in radiation environments could be improved by selecting the proper substrate materials, such as Al_2_O_3_, SiO_2_, Si_3_N_4_, SiC, and Si. Further work is needed to clarify it.
